# New LKB1 function in the primary cilium

**DOI:** 10.1186/2046-2530-4-S1-P71

**Published:** 2015-07-13

**Authors:** P Maurin, MJ Santoni, O Cabaud, S Marchetto, JP Borg, M Sebbagh

**Affiliations:** 1 F-13009, CNRS, Marseille, France; 2 F-13009, INSERM_CRCM_UMR_1068, Marseille, France; 3 F-13009, Intitut Paoli-Calmettes, Marseille, France; 4 F-13007, Université de la Méditerranée, Marseille, France

## 

The serine threonine kinase LKB1 is conserved and ubiquitously expressed throughout evolution. In humans, LKB1 is causally linked to the Peutz-Jeghers syndrome (PJS), an autosomal dominant inherited disorder characterized by melanocytic macules of the lips and multiple gastrointestinal hamartomatous polyps. PJS patients have a high risk of developing malignant tumours, including breast and gastrointestinal cancers. Moreover, LKB1 expression loss is frequently found in several cancer types such cervix, pancreas, or lung carcinomas which have led to classified LKB1 as a tumour suppressor. Mechanism(s) through which LKB1 exerts this tumour suppressor property remains an issue.

We and others have published results suggesting that the LKB1 complex is constitutively active in cells and that its regulation is in fact the result of its intracellular localization, allowing a spatiotemporal proximity with a subset of specific substrates. Although, LKB1 have been described to locate in the nucleus under ectopic expression, endogenous LKB1 appears to be mainly in cytosol, adherent junctions and primary cilium in polarized epithelial cells. LKB1 function(s) in cilia are still poorly understood even though involvement in mTOR repression has been proposed. Indeed, like for all proteins found in several cellular compartments, results from LKB1 inactivation is a mix of its functions loss in all compartments where its activity takes place impeding clear results for specific compartment.

Thus and through a new knock out mouse model which leads to specifically LKB1 activity and function loss in cilia, our work defines a new LKB1 function in this organelle which might be responsible, in part, for its tumour suppressor property.

Work supported by ARC association "Projet ARC 2011"; n: SFI20111203781.

